# Data on mixing of viscous fluids by helical screw impellers in cylindrical vessels

**DOI:** 10.1016/j.dib.2016.05.036

**Published:** 2016-05-25

**Authors:** Houari Ameur, Youcef Kamla, Abdessalam Hadjeb, Ilies Mohammed Arab, Djamel Sahel

**Affiliations:** a Institute of Science and Technology, University Center of Naâma (Ctr Univ Naâma), BP 66, 45000, Algeria; b Faculty of Technology, University Hassiba Ben Bouali of Chlef, Algeria; c Faculty of Mechanical Engineering, USTO-MB, BP 1505, El-M’Naouar, Oran 31000, Algeria

**Keywords:** Mixing, Helical screw agitator, Power consumption, Fluid circulation, Cylindrical tanks

## Abstract

In this article, the data assembled regarding the mixing of Newtonian and shear thinning fluids by screw impellers in a cylindrical tank is disclosed. The data summarizing some information on the efficiency of such impellers are obtained via 3D calculations of velocities and viscous dissipation in the whole vessel volume. The data presented herein may be useful for those who want to outline the mixing characteristics in terms of fluid circulation and power consumption for this kind of impellers, therefore, avoiding a great effort for achieving a high number of experiments.

**Specifications Table**

**Table:** 

Subject area	Chemical engineering
More specific subject area	Fluid mechanics
Type of data	Figure, Table
How data was acquired	Based on 3D calculations in the whole vessel volume
Data format	Analyzed
Experimental factors	The Glyerol syrup, Carpobol 940 and Natrosol solutions are used as working fluids to be mixed in the vessel.
Experimental features	The CFX tool is used to perform calculations and to solve the equations of energy and momentum. Calculations were achieved in a platform with Intel Core i7 CPU having clock speed of 2.20 GHz and 12.0 GB of RAM.
Data source location	Ctr Univ (Naâma) and USTO-MB (Oran), Algeria
Data accessibility	Data is provided in the article

**Value of the data**

• The data reveal the efficiency of screw impellers in cylindrical tanks.
• The described research is valuable for industrial processes involving homogenization of viscous fluids in mixing systems.
• The data provide information on the power consumption, flow velocities and fluid circulation for screw impellers with different blade pitch.

## 1 Data

In the present work, we provide the data generated on mixing of viscous Newtonian and non-Newtonian fluids by screw impellers in a cylindrical tank. We include four figures and two tables containing quantitative and qualitatitive information on the mixing characteristics.

## 2 Experimental design, materials and methods

### 2.1 Geometry studied

**Fig. 1:**
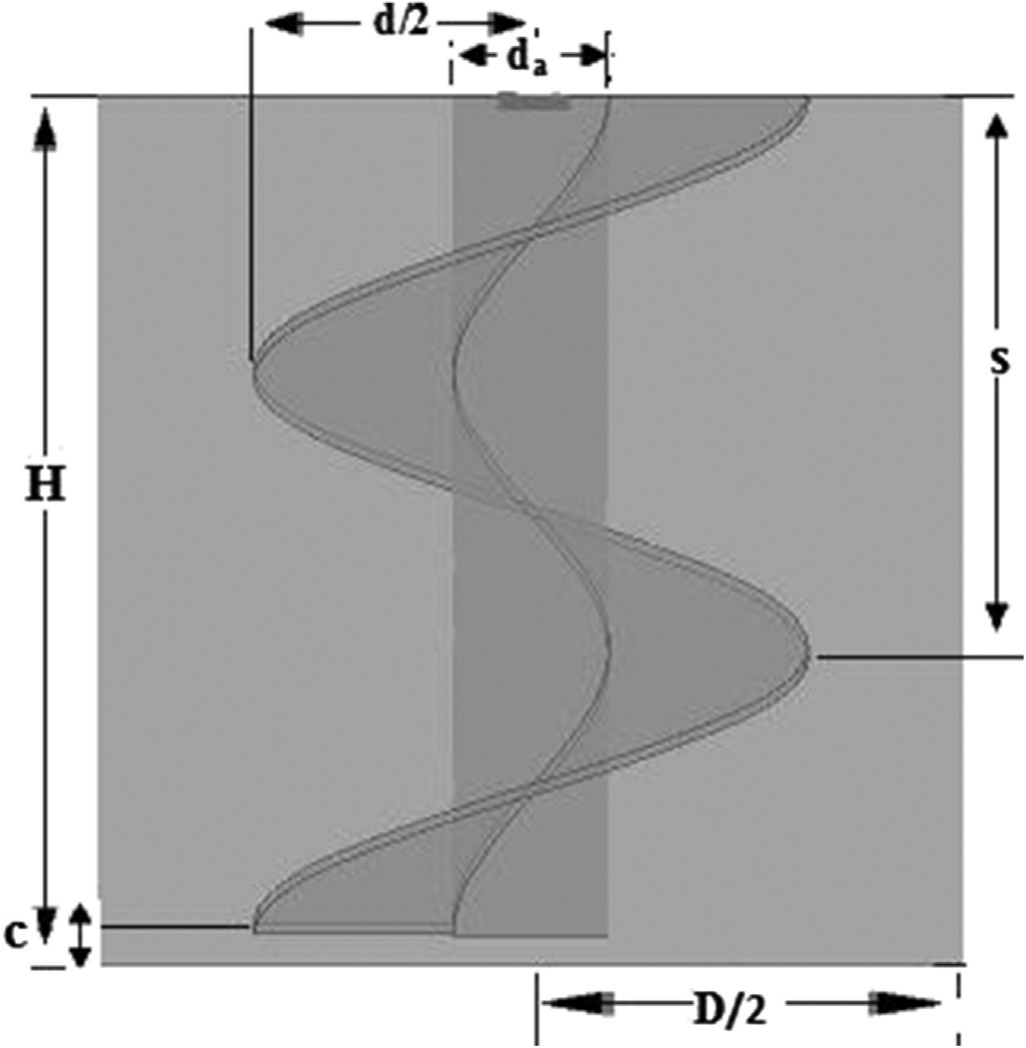
Configuration of the agitated tank.

The mixing system studied is shown in Fig. 1. It is a cylindrical tank agitated by a screw impeller. Details of all geometrical parameters are summarized in Table 1. Four geometries with different values of the pitch (*s*) are realized and which are: *s*/*D*=1.5, 1, 0.75 and 0.5, respectively. Mixing is operating in laminar flow for Reynolds numbers ranging between 0.1 and 30. Three liquids are used: a Glucose syrup, Carbopol 940 and Natrosol solution. Details on the rheological properties of the three liquids are given in Table 2. The fluid height (*h*) is equal to the tank height (*H*).

### 2.2 Mathematical equations

The power consumption (*P*) is calculated by integration of the viscous dissipation (*Q_v_*) in the whole vessel volume:

(1) $$P\;=\;\eta \;\int_{vessel\;\;volume}Q\;dv$$

where *η* is the viscosity. The dimensionless power number is given by the following equation:

(2) $$Np=\frac{P}{\rho N^{3}d^{5}}$$

where *N* is the rotational speed of the impeller and *ρ* is the density. For a shear thinning fluid (Ostwald model), the Reynolds number is defined as

(3) $$Re_{g}\;=\;\frac{\rho N^{2-n}d^{2}}{m}$$

The non-dimensional circulation number $N_{Qc}$ is also an important parameter calculated by the following equation:

(4) $$N_{Qc}=\frac{Q_{c}}{Nd^{3}}$$

where *Q_c_* is the circulation flow rate. Further details on the mathematical equations can be found in our previous paper [3].

### 2.3 Data obtained

**Fig. 2:**
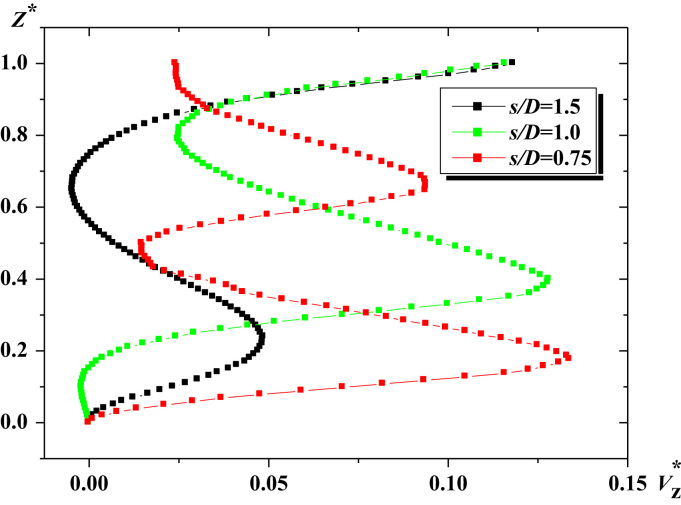
Axial velocity for the Natrosol liquid, *Re_g_*=10, R^*^=2*R*/*D*=0.5.

For a radial position near the blade tip (R^*^=2*R*/*D*=0.5), variations of the axial velocity $\left(V_{z}^{*}\;=V_{z}/\pi ND\right)$ along the vessel height (Z^*^=*Z*/*D*) are followed and presented in Fig. 2. As remarked, the velocities with high magnitudes are located at the blade tip. In a comparison between the three cases studied (*s*/*D*=0.75, 1 and 1.5), the lower values of $V_{z}^{*}$ are obtained for the case *s*/*D*=1.5, especially in the lower part of the vessel where the mixing is poor. When decreasing the pitch ratio, the agitated region is becoming wider.

**Fig. 3:**
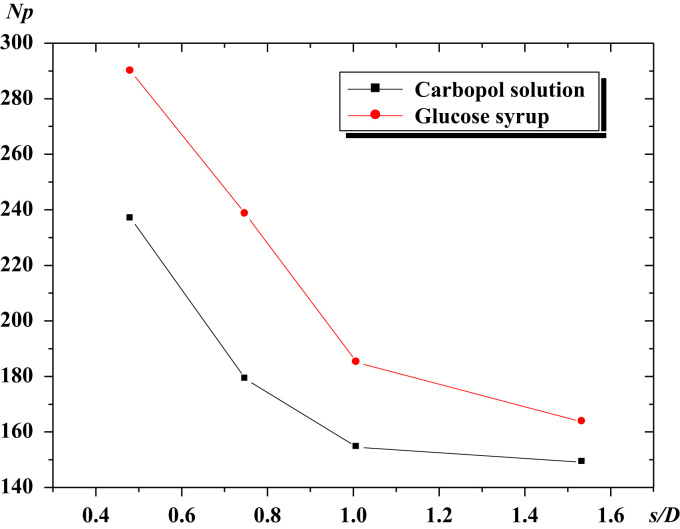
Power number vs. the blade pitch for different fluids.

The power consumption and fluid circulation are important parameters to determine the performance of a mixing system. The power number is calculated for different values of the blade pitch (*s*/*D*) and plotted in Fig. 3. The power required is found to be inversely proportional to the ratio (*s*/*D*), which is explained by the increase of the contact area between the impeller blade and the fluid.

**Fig. 4:**
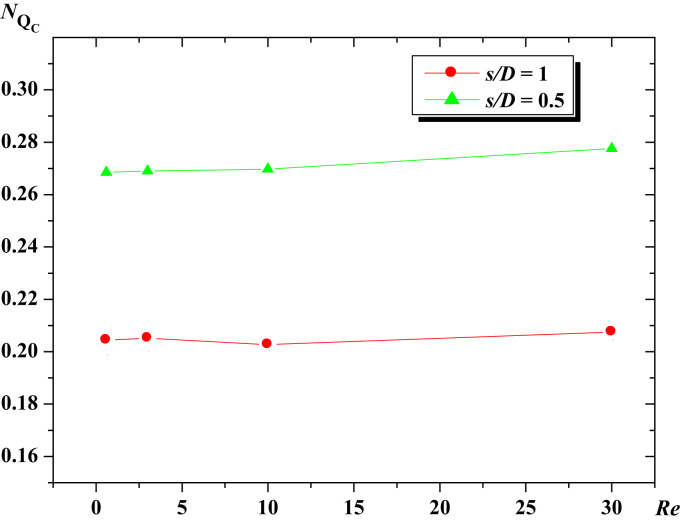
Circulation number vs. Reynolds number, for the Glucose syrup.

The obtained results for the circulation number (*N*_*Qc*_) are depicted in Fig. 4. *N*_*Qc*_ is found to be independent from Reynolds number (*Re*) in the flow region studied and it increases with the increase of the (*s*/*D*) ratio.

## 3 Data analysis

The data assembled is analyzed in Fig. 2, Fig. 3, Fig. 4.

## Figures and Tables

**Table 1:** Details on the geometrical parameters.

*D* [mm]	*H*/*D*	*d*/*D*	*c*/*d*	*d*_*a*_/*D*
400	1	0.64	0.06	0.18

**Table 2:** Rheological properties of all working fluids.

	Rheological behavior	Viscosity *μ* [Pa s]	Flow index *n* [dimensionless]	Consistency *m* [Pa s^*n*^]
Glucose syrup [1]	Newtonian	1	–	–
Carbopol 940 [1]	Shear thinning	–	0.22	8.39
Natrosol [2]	Shear thinning	–	0.59	10.8
